# Stabilization and activation of molecular oxygen at biomimetic tetrapyrroles on surfaces: from UHV to near-ambient pressure

**DOI:** 10.1039/d0na00827c

**Published:** 2021-02-01

**Authors:** Erik Vesselli

**Affiliations:** Department of Physics, University of Trieste Via A. Valerio 2 34127 Trieste Italy; CNR-IOM Area Science Park, S.S. 14 km 163.5 34149 Basovizza Trieste Italy evesselli@units.it

## Abstract

Recent advances in the development of surface science methods have allowed bridging, at least partially, the pressure gap between the ultra-high vacuum environment and some applicative conditions. This step has been particularly critical for the characterization of heterogenous catalytic systems (solid–liquid, solid–gas interfaces) and, specifically, of the electronic, structural, and chemical properties of tetrapyrroles at surfaces when arranged in 2D networks. Within a biomimetic picture, in which 2D metalorganic frameworks are expected to model and reproduce in a tailored way the activity of their biochemical proteic counterparts, the fundamental investigation of the adsorption and activation of small ligands at the single-metal atom reaction sites has progressively gained increasing attention. Concerning oxygen, biology offers a variety of tetrapyrrole-based transport and reaction pockets, as *e.g.* in haemoglobin, myoglobin or cytochrome proteins. Binding and activation of O_2_ are accomplished thanks to complex charge transfer and spin realignment processes, sometimes requiring cooperative mechanisms. Within the framework of surface science at near-ambient pressure (towards and beyond the mbar regime), recent progress has unveiled novel and interesting properties of 2D metalorganic frameworks and heterostacks based on self-assembled tetrapyrroles, thus opening possible, effective applicative routes in the fields of light harvesting, heterogenous (electro-)catalysts, chemical sensing, and spintronics.

## Abstract 2

Bridging the pressure gap in surface science has recently allowed the investigation *in situ* of the fundamental properties of biomimetic 2D metallorganic networks, relevant for the adsorption and activation of small strategic ligands like dioxygen.

## Graphical abstract

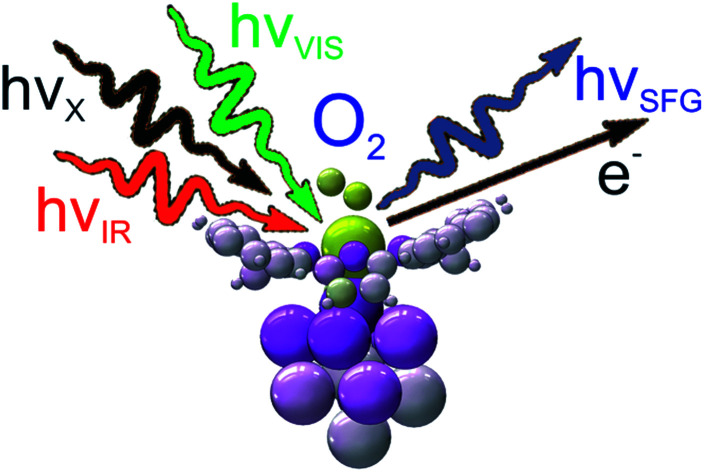

## 1. Introduction

Light harvesting and chemical conversion of energy vectors represent relevant technological issues that will deeply affect the future of our energy economy in the view of a sustainable scenario. The development of novel, nano-engineered materials would allow indeed the efficient and sustainable conversion and the storage of solar energy by exploiting several routes like *e.g.* water splitting and/or carbon dioxide activation and reduction to chemical energy vectors. Photosynthesis in Nature already provides a way to store sunlight in chemical forms by splitting water into oxygen and hydrogen. It's about a thermodynamically disfavoured process, with an energy cost of 2.46 eV.^1^ The combustion of H_2_ (or other hydrogen-containing chemical vectors) in fuel cells yields large amounts of free energy with only water and heat as side wastes, thus closing ideally the cycle. In the whole process, hydrogen and oxygen evolution reactions (HER and OER, respectively), and the oxygen reduction reaction (ORR) are key catalytic processes that actually determine the efficiency and selectivity of the conversion steps.^1^ Thus, a strategic milestone to achieve sufficient insight to design and development of tailored materials to this purpose is a full comprehension and an atomic-level, detailed description of the catalytic activation mechanisms of stable, small molecules like oxygen.^2–5^ In this sense, Nature has a lot to teach, since biological processes largely exploit O_2_ transportation and activation. A biomimetic approach may provide useful insight into reaction centres based on tetrapyrroles in the case of several reaction schemes,^6–8^ including O_2_ activation.^1,9–13^

### 1.1 From Nature to biomimetic heterostacks: molecular- and surface trans-effect

**Fig. 1:**
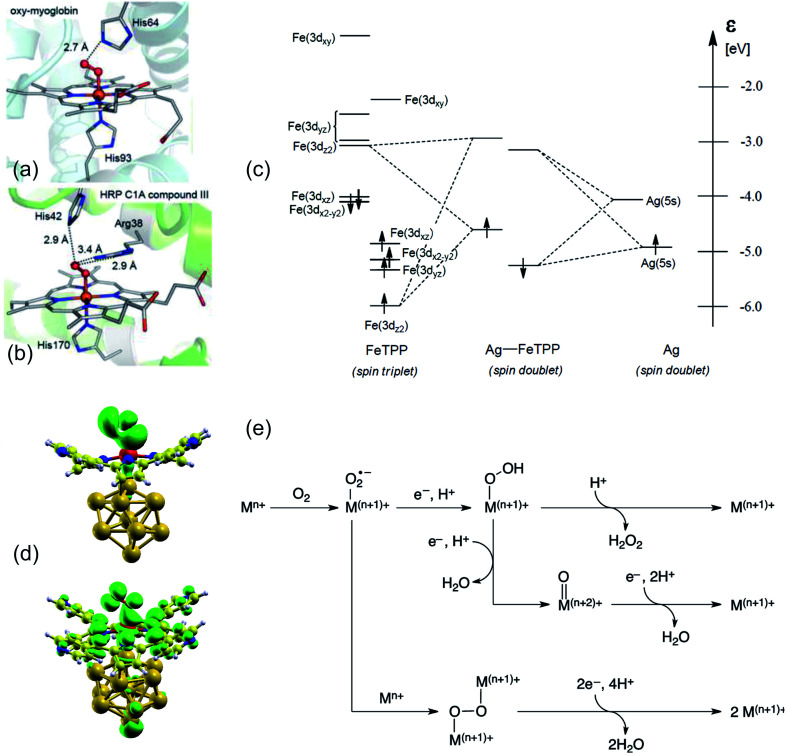
(a and b) Structure of ferric-superoxo intermediates of two selected, representative heme proteins that bind dioxygen: oxy-myoglobin (left) and the HRP C1A compound (right) [adapted with permission from ref. 12 – further permissions related to the material excerpted should be directed to the ACS]. (c and d) Surface trans-effect and rehybridization as biomimetic 2D counterparts of the molecular trans-effect: (c) qualitative energy diagram of the adsorption of a CoTPP on Ag [adapted with permission from ref. 14 – Copyright 2011 American Chemical Society] and (d) isodensity surfaces of the electronic states of the O_2_–CoTPyP/Au_13_ system [reprinted and adapted with permission from ref. 16]. (e) Two- and four-electron pathways for O_2_ reduction to H_2_O_2_ and H_2_O, respectively, catalysed by metal complexes [reprinted with permission from ref. 1 – Copyright 2017 American Chemical Society].

Binding and/or activation of small ligands in the biochemistry of enzymes occur by means of a combination of adsorption, charge transfer, and steric effects taking place in a reaction pocket where, in many (but of course not all) cases, a single metal ion is stabilized by a tetrapyrrolic matrix playing the major role (Fig. 1a and b).^10–12^ The charge and spin configurations of the metal ion, together with the organic coordination environment, determine the actual catalytic activity and selectivity of the system with respect to specific reactions. In this sense, a biomimetic strategy may consist in the synthesis of 2D, ordered heterostacks of porphyrin-based reaction centres supported by metal surfaces. The latter act both as geometric templates and as electron reservoirs.^10^ As we will discuss in detail in the following, the planar configuration of the tetrapyrrolic structure exposes for ligation both sides of the metal ion. In the case of biochemical systems, in addition to the effect of the residues that directly bind to the porphyrin's or corrole's macrocycle,^1^ the competition effects in the trans-coordination sphere between ligands yield tuning of the reaction centre's electronic configuration. This process, known as molecular trans-effect, corresponds to the surface trans-effect in the case of the biomimetic approach based on 2D crystalline systems (Fig. 1c).^7,8,14,15^ Indeed, the trans-effect describes in general the situation in which two ligands in *trans* position, binding to the same metal centre, compete for coordination.^14^ In the case of supported metallorganic molecules, one of the two ligands is replaced by the supporting surface. Thus, the latter mimics the biochemical trans-coordination counterpart, allowing for orbital rehybridization and charge transfer to the adducts across the porphyrin plane. It is indeed charge transfer in general, and electron donation in the specific case of the oxygen molecule, that actually contribute to the binding and activation of the ligand for further reaction (Fig. 1d).^9,16^

### 1.2 Oxygen stabilization and activation in Nature

The electronic ground state of O_2_ is a stable, low-reactivity triplet, which is 0.93 eV lower in energy with respect to the singlet state with paired spin configuration.^17^ In order to promote the molecule's activation for further reaction, biomimetic catalysts need to reproduce electron-donor sites. Here, the molecule can bind,^9^ electron donation can take place, and reactive superoxo (O_2_^−^) or peroxo (O_2_^2−^) species are formed, depending on the system.^18,19^ Similar charged metastable transition states were recently observed also at the surface of conventional ORR and OER electrocatalytic materials by means of *in situ* experimental approaches, confirming electron injection in O_2_ as one of the necessary steps for its activation.^20,21^ Surface reactive O_2_^−^, O^I−^, and O^II−^ species were identified by their associated O 1s core level and edge spectroscopic fingerprints.^20,22^ There is a long-lasting debate in the literature on this point, even for the “simple” case of oxygen interaction with single crystal metal surfaces in UHV,^23^ like for the Al(111) termination. In the latter case, competing oxygen–metal hybridization, spin selection rules, and charge transfer differentially contribute in the O_2_ adsorption/activation and dissociation mechanisms.^24–26^ Spin inversion is surely necessary for O_2_ activation by biosystems, like in heme. This is a key point since, even if ORR processes can be largely exothermic, spin-forbidden transitions contribute with high activation barriers. Most transition metals offer (i) unpaired electrons, both in the ground state or in almost degenerate excited states, allowing reaction with triplet O_2_, and (ii) spin–orbit coupled configurations, thus providing a quantum mechanical pathway to promote spin inversion,^17^ possibly exploiting diradical intermediates.^12^ The overall oxygen reduction process may involve two or four electrons (Fig. 1e):^1^
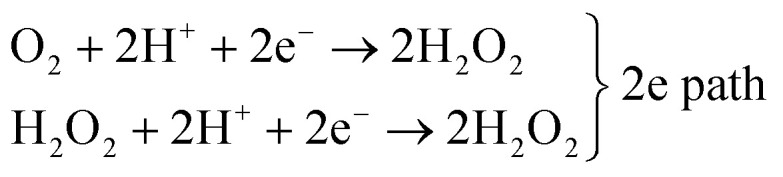
O_2_ + 4H^+^ + 4e^−^ → 2H_2_O, 4e path

The former pathway yields H_2_O_2_, a good reactant or possible energy carrier, and is generally mediated by late transition metal centres. The latter path is instead more relevant from the biological point of view, being also energetically more favoured (3.47 eV),^12^ and promoted by early transition metal complexes.^1^ The origin of these differences is explained on the basis of the selectivity towards the formation of terminal metal–oxygen intermediates as a result of the heterolytic cleavage of the O_2_ molecule. The d orbitals of late transition metal elements provide electrons that populate the metal-oxo antibonding states, thus making terminal oxo intermediates unstable. Nevertheless, in some systems, also late transition metals can catalyse four-electron O_2_ reduction processes, as in the case of bimetallic reaction centres. Already the very first one-electron step of oxygen reduction to the superoxide species is endoergonic, suggesting that a rigid, stepwise mechanism is rather unfavourable. Cooperative, multi-participated mechanisms are surely more effective, in which the tetrapyrrole centre plays several functions. This is for example the case of the oxygenation mechanism promoted by mono-oxygenase, where a two-electron process takes place. One e^−^ reduces the Fe(iii) ferric site to the Fe(ii) ferrous species and is subsequently transferred to the O_2_ adduct that binds forming a ferric-superoxo intermediate.^9^ A second e^−^ couples with a proton, yielding a ferric-(hydro)peroxo intermediate, promoting the heterolytic scission of the O–O bond by protonation of the distal oxygen atom. The electron transport chain is therefore a key step: in the case of heme proteins, the iron porphyrin sites are involved in both electron transport and ORR.^1^ In the specific case of Fe–O_2_ binding in heme complexes,^27^ a singlet electronic ground state is obtained upon reaction of triplet molecular oxygen with the high-spin Fe of the porphyrin (*S* = 2). The configuration of the complex is mainly explained on the basis of the Weiss model: a low-spin (*S* = 1/2) ferric centre is antiferromagnetically coupled to a doublet superoxide anion (O_2_^−^), where the Fe–O_2_ ligation is established by a σ(Fe–O) bond involving Fe 3d_*z*^2^_ and π*(O_2_) orbitals, and by a weak π bonding between Fe d_*yz*_ and π_⊥_*(O_2_).^12^ Not only Fe, but also Co plays relevant roles in the ORR. Cobalt–dioxygen ground states are doublets (*S* = 1/2) where a Co(iii) unpaired electron is donated to the O_2_ moiety. A Co(ii)–octaethylporphyrin supported on graphite was found to bind dioxygen (0.72 eV), as well as CoTPyP/Au(111) (0.85 eV) thanks to electron donation from the substrate, showing that a biomimetic approach can yield interesting results.^12,16^ Indeed, recent developments have shown that metal–organic frameworks (MOFs) based on tetrapyrroles and non-heme M/N/C (M = metal, N = nitrogen, C = carbon) catalysts may provide an optimal playground for oxygen activation.^28,29^ However, beyond the local electronic configuration, many other aspects need to be considered in order to tailor the active sites, since trans-effects,^14^ distortion of the tetrapyrrolic plane,^13^ electrostatic interactions,^12^ cooperative effects,^30^ and steric effects^31^ play relevant roles.

### 1.3 Tetrapyrroles at the solid–liquid interface

**Fig. 2:**
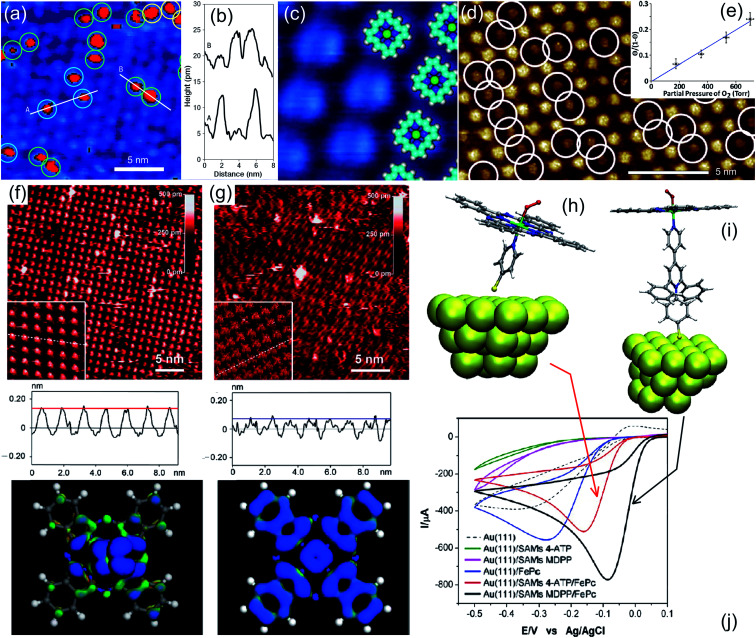
(a–c) STM imaging of a manganese porphyrin monolayer on Au(111) in *n*-tetradecane flushed with O_2_, where bright spots in (a) (8% of the centres) indicate dioxygen ligation; height profiles corresponding to cuts A and B are shown in (b), while (c) matches the filtered appearance of the molecules in the STM image with their ball models [adapted with permission from ref. 32]. (d and e) CoOEP/HOPG surface in phenyloctane saturated with O_2_ at room temperature (porphyrins with the ligand appear dimmer – white circles) and Langmuir O_2_ saturation curve [adapted with permission from ref. 33 – Copyright 2012 American Chemical Society]. (f and g) STM images and line profiles of FePc/Au(111) in 0.1 M HClO_4_ saturated with oxygen (f) or nitrogen (g); in the bottom panels the calculated DOS distributions are shown, corresponding to the LUMOs giving origin to the observed contrast in the STM imaging [adapted with permission from ref. 34 – Copyright 2106 American Chemical Society]. (h–j) Tuning the trans-effect for O_2_ ligation: MPc–L–Au_26_ simulation clusters with M = Fe, Cu and L = 4-ATP, MDPP (h and i) and corresponding voltammetry experimental results (j) [adapted with permission from ref. 37 – Copyright 2012 American Chemical Society].

Concerning biomimetic 2D systems based on tetrapyrroles, several efforts have been recently performed in order to characterize the oxygen interaction at the solid–liquid interface of electrolytic environments *in situ* and *operando* in real time.^32^ Specifically, the interest for the single-atom level detail is growing in order to achieve a thorough and fundamental insight. Scanning Tunnelling Microscopy (STM) is the main technique in this specific case, limited however to selected system choices and by environmental constraints. In this way, homolytic O_2_ cleavage was observed on Mn porphyrins on Au(111) in Ar-saturated *n*-tetradecane, yielding reactive Mn(iv)=O species (Fig. 2a–c).^32^ The reversible binding of O_2_ (0.71 eV) with Co(ii)OEP/HOPG molecules has been observed in a Langmuir uptake experiment in phenyloctane (Fig. 2d and e).^33^ Formation of the Fe–O_2_ complex was observed for the FePc/Au(111) system in 0.1 M HClO_4_ as a transition state of the ORR to H_2_O_2_*via* the two-electron mechanism occurring at 50 mV potential (Fig. 2f and g).^34^ In the same environment, also CoP and CoOEP dyes can stabilize oxygen and catalyse the ORR,^35,36^ at variance with CuTPP molecules.^36^ By further pushing the trans-effect through anchoring of the FePcs to Au(111) *via* 4-ATP or MDPP, a 4-electron process to yield H_2_O was instead observed (Fig. 2h–j).^37^ It was shown that, by exploiting trans-ligation, the O_2_–FePc bond strength can be tuned by a factor of about 3 (from 0.56 to 1.9 eV). Also bimetallic networks can be exploited, like the case of M^1^TPyP–M^2^ (M^1,2^ = Fe, Co) monolayers on Au(111), where cooperative mechanisms play a contributing role in the ORR in 0.1 M NaOH.^38,39^

### 1.4 Specific focus of this review

Despite the remarkable insight achieved at the liquid/solid interface thanks to *in situ* atomic resolution imaging approaches, still, the influence of the environment (very selected solvents and systems) and the lack of available complementary characterization techniques prevent a genuine focus on the fundamental aspects of the O_2_ adsorption and activation mechanisms. On the other way, Ultra-High Vacuum (UHV) methods typical of surface science approaches can provide deeper insight, but severely suffer from the extreme pressure gap with respect to ambient, biological, or applicative conditions.^8^ The recent development of spectroscopic techniques that allow at least a partial mitigation of the pressure gap without loss of insight opened to the possibility of investigating 2D systems up to Near-Ambient Pressure (NAP) conditions. These techniques include Near-Ambient Pressure X-ray Photoelectron Spectroscopy (NAP-XPS) and Infrared-Visible Sum-Frequency Generation spectroscopy (IR-Vis SFG).^8,40–43^ A number of papers have appeared, in which the UHV-like detailed characterization (Section 2.1) of the mechanisms involved in the adsorption (Section 2.1.1), activation (Section 2.1.2), and intercalation (Section 2.1.3) of ligands at well-defined, ordered 2D monolayers of tetrapyrroles was extended to the room temperature and NAP range (Section 2.2). This opened the way to the observation of novel phenomena, where not only adsorption (Section 2.2.1), but also reaction and gas-induced self-metalation processes (Section 2.2.2) take place.^44,45^ Thus, the latter active surfaces could be characterized *in situ* and *operando*.^7,8,16,44–50^ The review focusses on these recent advances, paying specific attention to the oxygen adsorption and activation mechanisms.

## 2. Solid–gas interfaces from UHV to near-ambient pressure: oxygen interaction with 2D metallorganic frameworks

Within the framework of ultra-high vacuum surface science, the adsorption of ligands on tetrapyrroles generally requires cryogenic temperatures. This is ascribed to the small binding energy of the ligands to the metal ions, of the order of a few tenths of an eV. As an example, adsorption of carbon monoxide or nitric oxide onto the FePc/Au(111) monolayer is observed at 20 K,^51,52^ while pyridine and ammonia bind at 80 K.^51^ NH_3_ was found to bind to NiTPP/Co(001) after an uptake performed at 78 K.^53^ However, nitrosyl complexes can be more stable in some cases and *e.g.* form in UHV on M(ii)–tetraphenylporphyrins (M = Fe, Co, Zn) on Ag(111) at 140 K,^14^ and even at room temperature on CoTPP/O/Ni(001)/Cu(001)^54^ and NiTPP/Cu(001).^55^ Apart from these few cases, the population of the reactive metal ion centres of tetrapyrroles with ligands at room temperature, thus at applicative conditions, requires pressure regimes beyond the conventional surface science environment, generally in the few mbar range, in line with the biologic counterparts. Recently, the first *in situ* experimental observation of the monocarbonylation of FePc/Ir(111) was obtained at room temperature in the mbar pressure range,^46^ thus opening to the possibility of investigating these systems at close-to-ambient conditions with atomic-level insight.^8^ Soon thereafter, the monocarbonylation of FePc/GR/Ir(111) at 10 mbar unveiled interesting singlet-fission mechanisms involved in the efficient absorption of visible light and its conversion in long-lived excitons.^44^ Carbon dioxide could be stabilized on the same system by means of a biomimetic tuning of the charge transfer through the metallorganic heterostack.^7^ A similar evolution in the fundamental characterization process could be observed in the very recent years also for the study of dioxygen adsorption.

### 2.1 Oxygen adsorption and activation in UHV

#### 2.1.1 Oxygen adsorption on 2D frameworks at surfaces in UHV

**Fig. 3:**
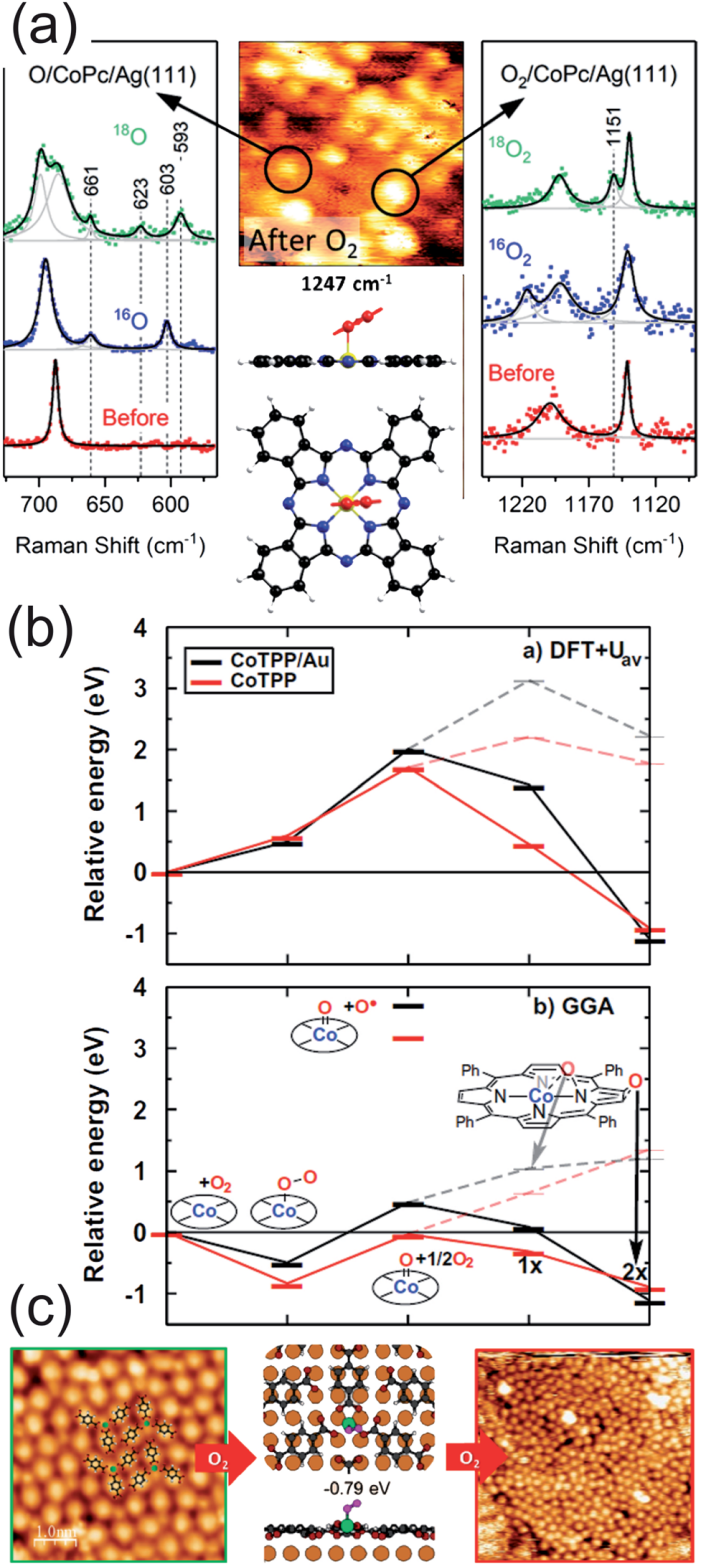
(a) TERS spectra, STM image, and computational results evidencing the partial stabilization of O_2_ ligands (right), undergoing then dissociation (left), on Co phthalocyanines on the Ag(111) surface in UHV [adapted with permission from ref. 56 – Copyright 2018 American Chemical Society]. (b) Functional-dependent DFT results for the possible stable adsorption of O_2_ at both supported (black) and unsupported (red) CoTPPs [reprinted with permission from ref. 57]. (c) STM and DFT-based evidence for O_2_ stabilization at a Mg-TPA network grown on Cu(100) [reprinted with permission from ref. 58 – further permissions related to the material excerpted should be directed to the ACS].

Partial stabilization of the dioxygen adduct was observed at room temperature on Co(ii)Pc/Ag(111) upon exposure of the layer to 1800 L of O_2_.^56^ The combination of Scanning Tunnelling Microscopy (STM), Raman spectroscopy, and Density Functional Theory (DFT) approaches allowed the identification of end-on O_2_/CoPc/Ag(111) species, always coexisting with the dissociation products, namely O/CoPc/Ag(111). In the STM images, bright asymmetric protrusions appear upon dioxygen ligation to Co, associated with the O–O tilting, at variance with the O/CoPc molecules (Fig. 3a middle-top). The O–O internal stretching mode is identified by isotopic labelling from TERS data combined with *ab initio* calculations (Fig. 3a). The tilted, end-on dioxygen stable ligation geometry at the Co atom is attained thanks to the surface trans-effect for which, upon adsorption on Ag(111), the Co atom of the CoPc receives more charge from both the porphyrin ring and the metal surface (0.98 e^−^) with respect to the gas phase tetrapyrrole case (0.77 e^−^). As already anticipated, also in the specific case of the CoPc/Ag(111) system it is evident that stabilization of adsorbed O_2_ requires charge donation, thus weakening the O–O bond and competing with dissociation through superoxo or peroxo intermediates, yielding the copresence of O/CoPc/Ag(111) species.

#### 2.1.2 Oxygen activation and dissociation

**Fig. 4:**
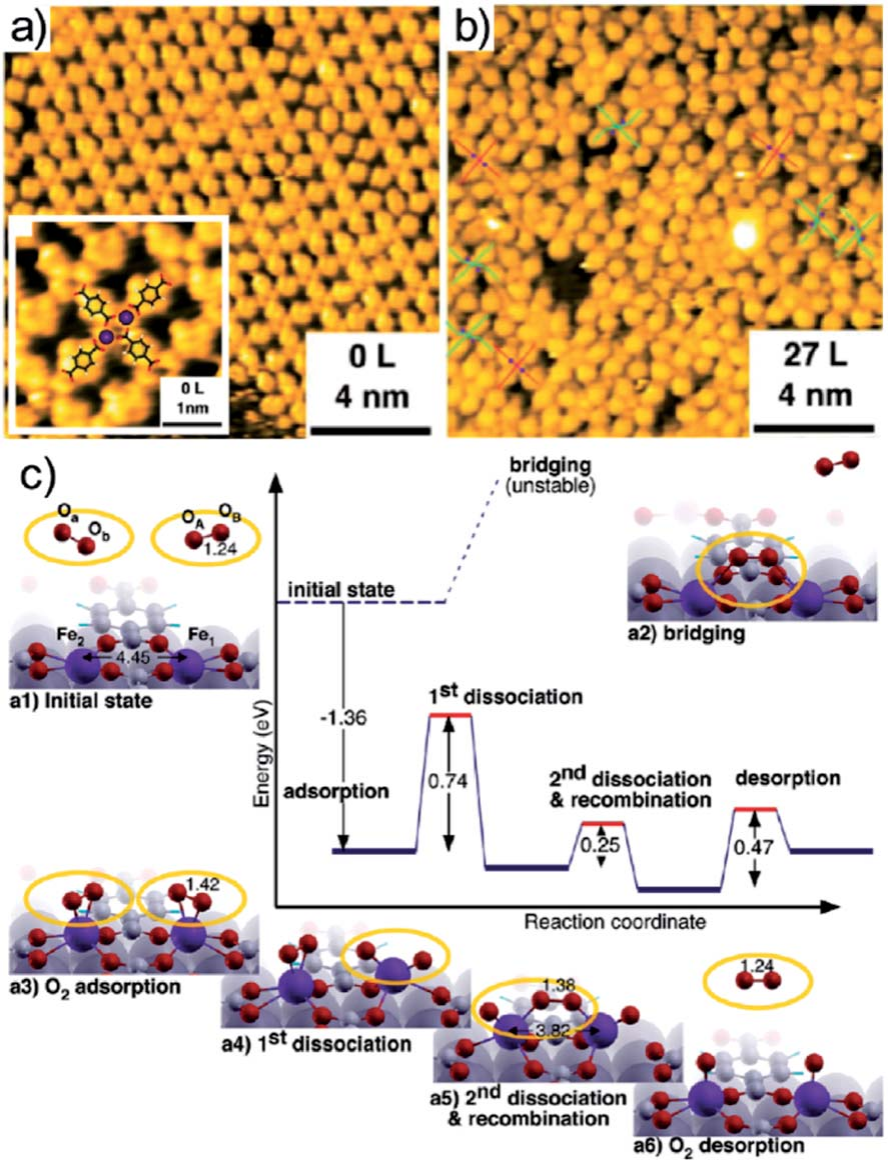
Oxygen adsorption and dissociation on di-iron TPA complexes on Cu(100): STM images before (a) and after (b) oxygen exposure and (c) reaction mechanism as predicted by means of DFT calculations [reprinted with permission from ref. 59 – Copyright 2011 American Chemical Society].

From the computational point of view, a detailed description of the O_2_ adsorption and possible decomposition at metal ions in tetrapyrroles is still an outstanding challenge, reflecting in the choice of the xc approximation within the DFT framework, in particular for large system sizes.^57^ As an example, for the case of O_2_ ligation to CoTPP, both in the gas phase and on Au(111), it is shown that the results obtained by means of approaches based on DFT + *U* and GGA can be dramatically different (Fig. 3b).^57^ In the latter case, stabilization of dioxygen is favoured and the dissociation barrier is of the order of 1 eV, while in the former case adsorption is energetically uphill and the O–O bond scission costs about 2 eV. Considering that the experimental evidence proves that, for the CoPc/Ag(111) system, stabilization and activation pathways may coexist (Fig. 3a),^56^ this makes the picture quite puzzling. An even more complex example is represented by the interaction of O_2_ with a Mg-TPA network on Cu(100), a 2D biomimetic model of the RuBisCO enzyme active site.^58^ Interaction of dioxygen with the Mg^2+^ centre at room temperature occurs in UHV already after only 27 L O_2_, yielding the progressive disassembly and collapse of the network for larger exposures (Fig. 3c). A metastable dioxygen adduct binds to Mg^2+^ (0.79 eV) undergoing dissociation and inducing oxidation of both Mg and the uncovered Cu(100) substrate. The same TPA molecules on Cu(100) can self-assemble in order to coordinate di-iron sites, forming an ordered 2D superstructure (Fig. 4a),^59^ where O_2_ readily dissociates at room temperature in UHV (Fig. 4b). The complex mechanism reveals the cooperative contribution of adjacent Fe sites and multiple O_2_ molecules, with an activation barrier of only 0.74 eV (Fig. 4c). Also in this case, exposure to molecular oxygen progressively destroys the superstructure (Fig. 4b). Cooperative dissociation of O_2_ is observed for Mn(ii)TPP/Ag(111), where exposure of the Mn(ii)TPP monolayer yields adjacent couples of Mn(ii)OTPP molecules.^60^ Interestingly, oxygen activation and dissociation was observed in UHV also on metal-free, N-doped graphene grown on Ir(111). The system was exposed to 5 × 10^−5^ mbar of molecular oxygen at 200 °C for 30 min, inducing the formation of epoxy and ether species, accompanied by minority carbonyl species.^61^

#### 2.1.3 Oxygen at the interface

**Fig. 5:**
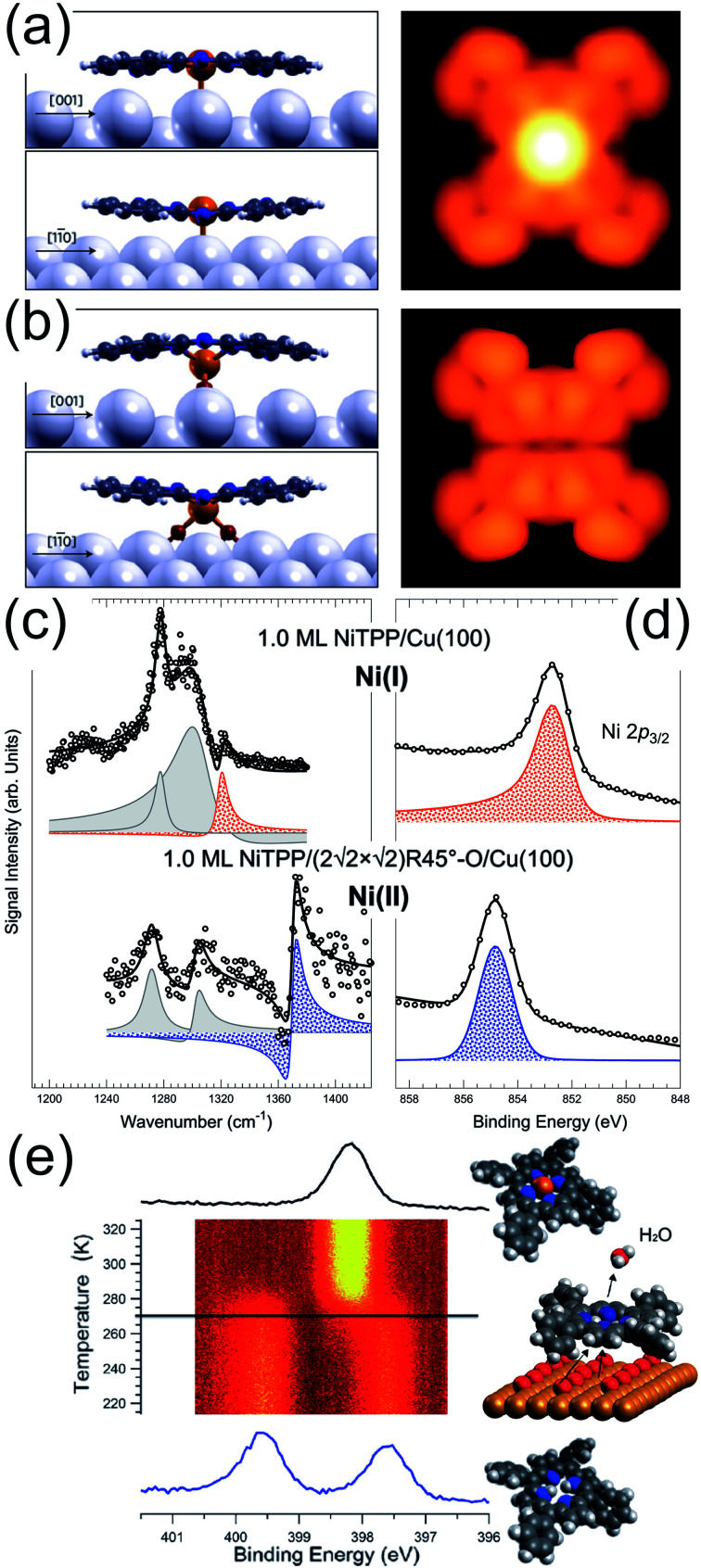
(a) R1-LD FePc/Ag(110) and (b) FePc–(η^2^-O_2_)–Ag(110) phases simulated by means of DFT [reprinted with permission from ref. 62]. (c) IR-Vis SFG and (d) Ni 2p_3/2_ XPS core level spectra of 1.0 ML NiTPP/Cu(100) without (top) and with (bottom) oxygen at the interface, stabilizing Ni(i) and Ni(ii) species, respectively.^45^ (e) N 1s core level evolution during the temperature-induced self-metalation of 2H-TPP/Cu(111) promoted by pre-adsorbed oxygen in UHV [reprinted with permission from ref. 69].

As discussed in the precious section, following exposure to O_2_, a few, selected metalorganic frameworks and tetrapyrroles can stabilize the dioxygen moiety and induce its dissociation also in UHV. However, while the O atoms can oxidize the metal ion, they may also interact with the underlying supporting surface. This is the case of the FePc/Ag(110) system.^62^ A number of ordered FePc superstructures can be grown on the Ag(110) termination in the (sub)-monolayer coverage regime (R1-LD, R2-LD, and O-HD). The R1-LD phase is a rectangular *c*(10 × 4) structure in which FePc molecules coordinate to on-top Ag sites. Upon exposure to 1700 L O_2_ at RT, the Fe centres of the tetrapyrroles undergo strong dimming in their STM imaging appearance (Fig. 5a and b). By combining experimental information and DFT modelling, it is demonstrated that oxygen intercalates at the tetrapyrrole–surface interface, forming a stable FePc–(η^2^-O_2_)–Ag coordination complex with a saddle-shape, distorted macrocycle (Fig. 5b). Interface oxygen can strongly influence the oxidation state of the coordinated metal and can be thus exploited as a chemical switch to control the metal ion reaction site. This occurs for the NiTPP/Cu(100) system.^45,63^ The strong metal–substrate interaction induces a sizeable charge transfer to the molecule, yielding population of the LUMOs up to the 3+ level and the formation of a Ni(i) ion, at variance to the Ni(ii) oxidation state typical of the gas phase porphyrin. On the contrary, in the NiTPP/O/Cu(100) layer, oxygen decouples the molecule from the substrate and the Ni(ii) oxidation state is preserved. The latter phenomena were observed at room temperature in UHV by means of a combination of spectroscopy, microscopy, and computational approaches (Fig. 5c and d). Finally, the combined presence of a supporting and interacting surface and of an oxygen phase is known to lower the metalation barrier for adsorbed tetrapyrroles. In the biologic environment, free-based species undergo metalation in the homogenous liquid phase by reaction with dissolved metal salts. Enzymes catalyse the process by a multi-step reaction pathway involving a significant geometric distortion of the macrocycle. At surfaces, at least two common aspects are shared with the biologic counterpart. The so-called sitting atop complex plays a relevant role as an intermediate metalation step,^64,65^ in which the metal centre already forms a coordination bond to the macrocycle, but the central H atoms are not yet released. Secondly, the molecule–surface interaction may induce geometric distortions of the tetrapyrrolic macrocycle, in analogy to the enzymatic process.^31,66^ This is obtained thanks to the conformational adaptation of the tetrapyrrole to the local adsorption environment through the flexibility of the porphyrin plane and the rotational degrees of freedom of the meso-groups.^67^ All these steps are facilitated by the co-presence of oxygen, finally forming water upon reaction with the central H atoms in the tetrapyrrole pocket.^8,68,69^ This is the case for the self-metalation reaction of 2H-TPP molecules adsorbed on Cu(111), where the pre-oxidation of the metal termination lowers the reaction temperature by 185 ± 15 K, down to room temperature (Fig. 5e).^69^ Same reasoning applies to the Pd(100) substrate, where the temperature-induced self-metalation of 2H-TPP to PdTPP cannot be obtained up to 600 K in UHV, when competing desorption and decomposition of the tetrapyrroles set in. Instead, by depositing the 2H-TPP molecules on the oxygen-saturated Pd(100) surface, full metalation of the layer can be accomplished already at 430 K.^48^

### 2.2 Oxygen adsorption and activation at NAP

By progressively increasing the oxygen pressure beyond the UHV limits and approaching the Near-Ambient Pressure (NAP) regime, new phenomena set in, as already evidenced for other small molecules like CO and CO_2_.^8^ We will discuss here two specific cases, *i.e.* oxygen binding, stabilization, and activation at Co tetrapyrroles on Au(111),^16^ and the oxygen-induced self-metalation of 2H-TPP on Pd(100).^49^

#### 2.2.1 Binding of molecular oxygen at near-ambient pressure

**Fig. 6:**
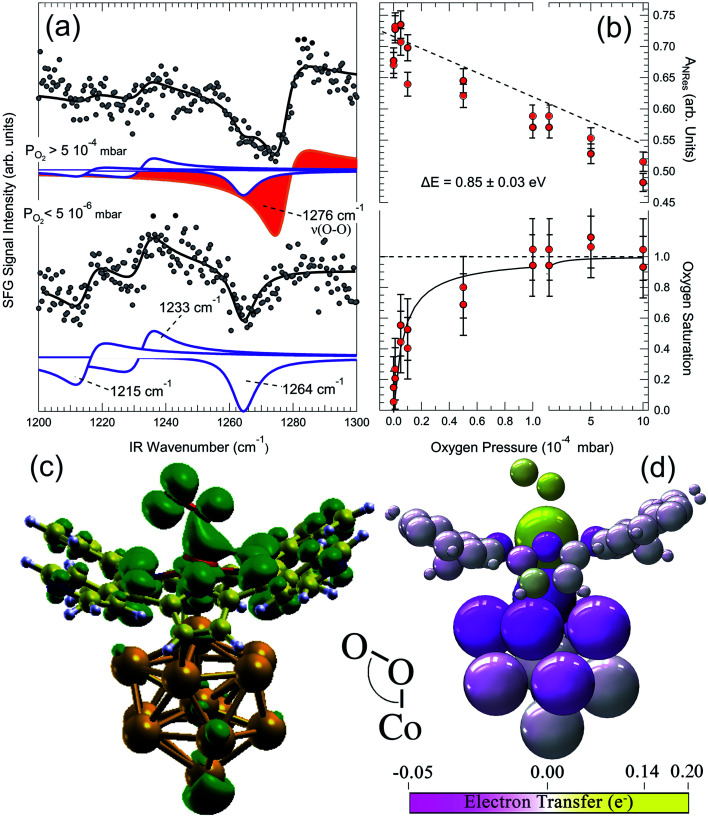
(a) IR-Vis SFG spectra of the pristine CoTPyP/Au(111) layer *in vacuo* (bottom) and in oxygen atmosphere (top) evidencing the growth of a vibronic resonance at 1276 cm^−1^, associated with the O–O stretch of the dioxygen ligand. (b) Evolution of the non-resonant signal (top) and O_2_ saturation curve (bottom) as a function of the oxygen background pressure obtained from the IR-Vis SFG spectra collected *in situ*. (c) Computed O_2_–CoTPyP/Au_13_ structural model and isodensity surface (green envelopes) of the state at 0.4 eV below the Fermi level: one of the macrocycle's N atoms contributes directly in the O_2_ bonding, while the angle originates from the alignment of the π*(O_2_) with the Co 3d_*z*^2^_. (d) Charge transfer occurring upon O_2_ ligation to the CoTPyP/Au_13_ system.^16^

A 2D metal–organic framework self-assembled at the Au(111) termination is able to mimic the O_2_ stabilization and activation mechanisms that are typical of the biochemical environment of proteins and enzymes.^16^ A CoTPyP/Au(111) layer binds indeed dioxygen at room temperature above 5 × 10^−4^ mbar, forming a covalent bond at the Co centre. Vibronic measurements performed *in situ* at O_2_ saturation conditions reveal the development of an O–O stretching mode at 1276 cm^−1^ (Fig. 6a), associated with a ligand configuration laying between the oxo- and the superoxo-species (O_2_^*δ*−^, *δ* = 0.22 *e* from DFT calculations for the specific case). The injection of charge into the ligand occurs *via* the surface trans-effect, from Au through the macrocycle, yielding both stabilization of the adduct and the weakening of its internal O–O bond. The O_2_–Co desorption energy is evaluated in a Langmuir uptake experiment (Fig. 6b) on the basis of a simple kinetic model. With the constrain of the strong assumption of a pre-exponential factor of 10^13^ s^−1^, a value of 0.85 ± 0.03 eV is obtained, compatibly with what obtained by *ab initio* calculations within the framework of DFT. Upon adsorption, O_2_ develops a strong dipole moment (0.11 D) due to the differential charging of the two non-equivalent O atoms, associated with the end-on bonding geometry. As in the biological counterpart, the O_2_ molecule sits on-top of the Co atom in an end-on configuration, and the molecular axis is tilted by 118° (Fig. 6c and d). There is evidence of a Co(3d_*z*_2)–O(2p) overlap yielding covalent bonding at −7.9, −1.0, and −0.4 eV below the Fermi level, with a strong contribution associated with the charge transfer from Au through the macrocycle plane (trans-effect) (Fig. 6d) and the formation of a shared Au–Co–O_2_ peak in the DOS at −7.9 eV. By comparing geometric and electronic structures, it is found that the Co–O_2_ bond consists of a dative σ(Co–O) interaction between vacant 3d_*z*^2^_ and π*(O_2_) and a weak π interaction between 3d_*yz*_ and π_⊥_*. The tilting originates from the geometry of the π*(O_2_), which, in order to vertically align with the Co 3d_*z*^2^_, requires the bending of the molecular axis (Fig. 6c, DOS isodensity surface at 0.4 eV below the Fermi level). Interestingly, it can be noticed that also one of the N atoms of the macrocycle participates directly in the oxygen bonding. The total O_2_ magnetization is lowered from S = 1 by 15% upon adsorption at the Co site. This is a key point in the activation process of O_2_: indeed, its ground state is a triplet, poorly reactive with other small, common molecules that are instead in a singlet state. As described in Section 1.2, activation involves the formation of superoxo or peroxo species, undergoing the 2e^−^ or the 4e^−^ reduction processes, depending on the system. Spin inversion is necessary since spin-forbidden transitions contribute with high activation barriers. Most transition metals offer unpaired electrons, both in the ground state or in almost degenerate excited states, allowing reaction with triplet O_2_, and spin–orbit coupled configurations, thus providing a quantum mechanical pathway to promote spin inversion, possibly exploiting di-radical intermediates for reaction.

#### 2.2.2 Molecular oxygen inducing self-metalation at near-ambient pressure

**Fig. 7:**
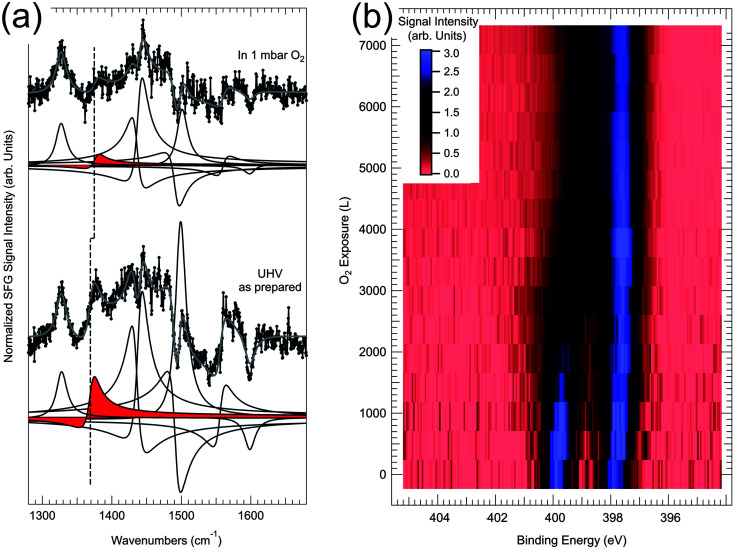
(a) IR-Vis SFG spectra of the pristine 2H-TPP/Pd(100) monolayer in UHV (bottom) and in 1 mbar O_2_ at room temperature (top). (b) NAP-XPS map showing the evolution of the N 1s core level upon exposure of the 2H-TPP/Pd(100) monolayer to oxygen at room temperature (the signal intensity is mapped in arbitrary units following the colour scale represented in the inset).^49^

As discussed above, the ordered 2H-TPP/Pd(100) monolayer can undergo self-metalation in UHV when atomic oxygen is pre-adsorbed at the interface and by heating to 430 K.^48^ Oxygen atoms favour the formation of the sitting-atop complex by lowering the metalation barrier, so that the reaction can be thermally activated below the critical temperature of competing 2H-TPP desorption and decomposition processes. However, it has been recently proven that by exposing the 2H-TPP/Pd(100) monolayer to 1 mbar O_2_ the self-metalation reaction to PdTPP can be induced already at room temperature.^49^ Evidence has been obtained by means of *in situ* IR-Vis SFG measurements (Fig. 7a). The intensity ratio of the vibronic resonances at 1327 and 1369 cm^−1^ was observed to be a good metalation marker when the 2H-TPP monolayer is deposited on the oxygen pre-covered Pd(100) termination and subsequently annealed to 410 K, thus inducing metalation and water production. By extending the investigation beyond UHV conditions, it is found that in 1 mbar O_2_ the feature at 1369 cm^−1^ undergoes a blue shift of 6 cm^−1^ and almost disappears (red deconvolution and dashed line in Fig. 7a). Three modes contribute to the resonance: the C_*β*_–C_*α*_–NH asymmetric stretching, the H rocking of the macrocycle pyrrolic moieties, and the C_*α*_–N–C_*α*′_ stretching. On the contrary, the lowest energy feature (1327 cm^−1^) mainly originates from the phenyl H rocking modes. The spectroscopic information suggests therefore a strong local deformation of the C–N bonds and the loss of the central H atoms, compatibly with the 2H-TPP metalation to PdTPP. NAP-XPS data of the N 1s core level confirm the picture. In the pristine layer in UHV, before exposure to oxygen, two symmetric N 1s spectral components of equal intensity can be resolved at binding energies of 399.8 and 397.8 eV, associated with the pyrrolic (N–H) and iminic N, respectively, of the 2H-TPP molecules. By exposing the layer to oxygen at room temperature, a dramatic evolution of the spectral lineshape is observed (Fig. 7b). Two intense N 1s photoemission peaks grow at 397.5 and 398.2 eV at the expenses of the former doublet, indicating full metalation and, additionally, allowing distinction between intact PdTPP porphyrins and PdTPP whose phenyl groups have undergone partial dehydrogenation due to reaction with adsorbed oxygen. By means of a simple kinetic model, the analysis of the dynamic uptake data of both IR-Vis SFG and NAP-XPS allow the quantitative evaluation of the self-metalation activation barrier (0.4 eV). Within this picture, the impinging O_2_ molecules undergo dissociation at the centre of the tetrapyrroles, contributing at the same time to the formation of the metalation transition state (first O atom, sitting-atop complex) and to the oxidation (second O atom) of the underlying Pd surface.

## 3. Summary and perspectives

I have reviewed very recent experimental steps towards the characterization of the interaction mechanisms of dioxygen with 2D heterostructures based on tetrapyrroles or MOFs in general. Important pieces of fundamental information have already been understood. Indeed, the cooperative contribution of several effects is necessary in order to stabilize and activate O_2_ at single metal atom sites. These include (i) the rehybridization of the O_2_–metal electronic states, (ii) the trans-effect yielding charge transfer to the ligand through and from the macrocycle, and (iii) the spin reconfiguration to rearrange the stable oxygen triplet. Biomimetic systems can be designed and tuned to achieve all these steps, but still there is something missing with respect to the biologic counterpart. Specifically, enzymatic and, more generally, proteins sites available for O_2_ binding and/or reaction are placed within 3D pockets. The 3D environment allows for (i) the control of the in and out flux of both reactants and products,^70^ (ii) the induction of steric/geometric effects,^31,71,72^ and (iii) the distal coordination of the ligands to functional terminations of the protein backbone.^73^ Thus, the biomimetic modelling of the functions and properties connected with the 3D nature of the binding pockets is still far from reach in the context of 2D heterostructures. Steps (i) to (iii) may represent the next challenges in the perspective of the development of novel 2D materials based on tetrapyrroles with applicative potential in the fields of heterogeneous (electro)-catalysis, light harvesting, electronics and spintronics.

## Conflicts of interest

There are no conflicts to declare.

## Supplementary Material
